# 

**Published:** 1883-03

**Authors:** 


### Pamphlets.

The Placental Development in Mammals. A Unity of Anatomical and Physiological Modality in all Vertebrates. By Henry O. Marcy, M.D. Boston. Reprint from *Annals of Anatomy and Surgery.*

The Physiology of Alcoholics. An address by William B. Carpenter, M.D., L L.D., F.R.S. New York: National Temperance Society and Publication House.

The management of chronic Inebriates and Insane Drunkards. By Albert W. Blodgett, M.D., Boston.

